# Supplementary material for: Post-acute sequelae of COVID-19 symptom phenotypes and therapeutic strategies: A prospective, observational study
Source: PLoS One. 2022 Sep 29;17(9):e0275274. doi: 10.1371/journal.pone.0275274 (PMC9521913; doi:10.1371/journal.pone.0275274)
Supplement: S1 Table — Subjects were asked 12-months after hospitalization for COVID, “Do you believe you have "post-COVID syndrome" also called "long hauler" syndrome or did you continue to have symptoms for at least 4 weeks after your initial diagnosis of COVID-19?” If the response was “yes”, they were presented with the options shown in this table. (DOCX) [file pone.0275274.s001.docx]

**Supplemental Table 1:** Symptom Questionnaire. Subjects were asked 12-months after hospitalization for COVID, “Do you believe you have "post-COVID syndrome" also called "long hauler" syndrome or did you continue to have symptoms for at least 4 weeks after your initial diagnosis of COVID-19?” If the response was “yes”, they were presented with the options shown in this table.

| **Question** | **Response** |
| --- | --- |
| **What are your prolonged COVID symptoms? (check all that apply). Note that symptoms must be present for at least 4 weeks following initial COVID diagnosis** | Yes/no |
| Brain fog/confusion/difficulty concentrating/memory loss | Yes/no |
| Headache | Yes/no |
| Shortness of breath | Yes/no |
| Fatigue | Yes/no |
| Muscle pain/ache | Yes/no |
| Joint pain/ache | Yes/no |
| Cough | Yes/no |
| Wheezing | Yes/no |
| Dizziness/lightheadedness | Yes/no |
| Irregular heartbeat or racing heart | Yes/no |
| Vision abnormalities (for example seeing spots, stars, lines, flashing lights, zigzag lines, heat waves or tiny dots on a gray, white or black background sometimes referred to as "snow") | Yes/no |
| Difficulty sleeping | Yes/no |
| Fever | Yes/no |
| Anxiety | Yes/no |
| Depression/sadness | Yes/no |
| Persistent loss of taste/smell | Yes/no |
| Post-exertional malaise | Yes/no |
| Fainting/blackouts | Yes/no |
| Lumpy toes (COVID toes) | Yes/no |
| Difficulty urinating | Yes/no |
| Tremors | Yes/no |
| Loss of appetite | Yes/no |
| Slowness of movement | Yes/no |
| Stiffness of muscles | Yes/no |
| Difficulty swallowing | Yes/no |
| Problems with balance | Yes/no |
| Loss of hearing | Yes/no |
| Ringing in the ears | Yes/no |
| Jerking of the limbs | Yes/no |
| Weakness of arms or legs | Yes/no |
| Other | Specify |
