# Supplementary material for: Post-acute sequelae of COVID-19 symptom phenotypes and therapeutic strategies: A prospective, observational study
Source: PLoS One. 2022 Sep 29;17(9):e0275274. doi: 10.1371/journal.pone.0275274 (PMC9521913; doi:10.1371/journal.pone.0275274)
Supplement: S2 Table — Subjects were asked, “What treatments have you used to deal with your prolonged COVID symptoms? Please check left column if ever used, and also right column if therapy helped post-COVID symptoms”. (DOCX) [file pone.0275274.s002.docx]

**Supplemental Table 2:** Therapy Questionnaire. Subjects were asked, “What treatments have you used to deal with your prolonged COVID symptoms? Please check left column if ever used, and also right column if therapy helped post-COVID symptoms”

| **Therapy Type** | **Ever Used?** | **Helped symptoms?** |
| --- | --- | --- |
| Physical Therapy | Yes/no | Yes/no |
| Occupational Therapy | Yes/no | Yes/no |
| Psychiatric/Psychologist/Talk therapy | Yes/no | Yes/no |
| Stimulants (e.g. Modafanil/provigil, ritalin, adderall, etc) | Yes/no | Yes/no |
| Sleep medications (e.g. melatonin, ambien, benadryl, Lunesta etc) | Yes/no | Yes/no |
| Antidepressants | Yes/no | Yes/no |
| Anti-anxiety medications (SSRI, SNRI, buspar etc) | Yes/no | Yes/no |
| Pain medication (e.g. NSAIDs, tylenol etc) | Yes/no | Yes/no |
| Inhalers | Yes/no | Yes/no |
| Steroids | Yes/no | Yes/no |
| Chiropractor | Yes/no | Yes/no |
| Meditation | Yes/no | Yes/no |
| Acupuncture | Yes/no | Yes/no |
| Other | Specify | Specify |
