# Supplementary material for: Post-acute sequelae of COVID-19 symptom phenotypes and therapeutic strategies: A prospective, observational study
Source: PLoS One. 2022 Sep 29;17(9):e0275274. doi: 10.1371/journal.pone.0275274 (PMC9521913; doi:10.1371/journal.pone.0275274)
Supplement: S3 Table — Subjects were asked “Have you experienced any of the following stressors within the last month? (check all that apply in the past month)”. (DOCX) [file pone.0275274.s003.docx]

**Supplemental Table 3:** Stressor Questionnaire. Subjects were asked “Have you experienced any of the following stressors within the last month ? (check all that apply in the past month)”.

| **Stressor** | **Response** |
| --- | --- |
| Social Isolation | Yes/no |
| Financial Insecurity | Yes/no |
| Food Insecurity/lack of food | Yes/no |
| Homelessness | Yes/no |
| Domestic violence/abuse | Yes/no |
| Relationship problems with member(s) your household | Yes/no |
| Education disruption (for yourself or a member of your household) | Yes/no |
| Increased caregiver responsibilities | Yes/no |
| Personal illness | Yes/no |
| New disability | Yes/no |
| Death of family member/friend | Yes/no |
| Illness of family member/friend | Yes/no |
| Unemployment | Yes/no |
| Lack of access to childcare | Yes/no |
| Political conflict with family/friends/colleagues | Yes/no |
| Other | Specify |
