# Supplementary material for: Post-acute sequelae of COVID-19 symptom phenotypes and therapeutic strategies: A prospective, observational study
Source: PLoS One. 2022 Sep 29;17(9):e0275274. doi: 10.1371/journal.pone.0275274 (PMC9521913; doi:10.1371/journal.pone.0275274)
Supplement: S4 Table — (DOCX) [file pone.0275274.s004.docx]

**Supplemental Table 4.** Univariate odds ratios and 95% confidence intervals representing the association of demographics, comorbidities, symptom clusters and 12-month outcome metrics with Therapy Groups (N=122 PASC patients).

|  | Therapy group A: (N=72)  Received few therapeutic interventions, most often exercise and pain medications  OR, 95% CI, P | Therapy group B: (N=12)  Received several interventions, most notably psychological talk therapy, anti-depressants, anti-anxiety medications and physical therapy  OR, 95% CI, P | Therapy group C: (N=38)  Primarily received physical and occupational therapy  OR, 95% CI, P |
| --- | --- | --- | --- |
| **Demographics** |  |  |  |
| Age, median (IQR) | **0.96 (0.93-0.99) P=0.009** | 1.04 (0.99-1.09) P=0.159 | 1.03 (1.00-1.07) P=0.054 |
| Sex (male) , N (%) | 1.11 (0.53-2.33) P=0.780 | 0.41 (0.12-1.37) P=0.147 | 1.31 (0.59-2.91) P=0.511 |
| Race (white) , N (%) | 0.86 (0.38-1.94) P=0.712 | 2.02 (0.51-7.99) P=0.317 | 0.87 (0.37-2.06) P=0.751 |
| Education level >12 years, N (%) | 1.77 (0.50-6.25) P=0.377 | 0.81 (0.09-7.19) P=0.850 | 0.57 (0.14-2.24) P=0.416 |
| **Comorbidities** |  |  |  |
| Pre-COVID disability (mRS) , N (%) | 0.84 (0.61-1.15) P=0.278 | 0.78 (0.39-1.55) P=0.475 | 1.29 (0.94-1.77) P=0.119 |
| Hypertension, N (%) | 0.60 (0.28-1.26) P=0.176 | 1.25 (0.37-4.20) P=0.718 | 1.62 (0.74-3.55) P=0.228 |
| Diabetes, N (%) | 0.61 (0.27-1.37) P=0.229 | 0.93 (0.24-3.68) P=0.919 | 1.78 (0.77-4.13) P=0.180 |
| COPD/Asthma, N (%) | 1.29 (0.40-4.09) P=0.671 | 1.63 (0.32-8.36) P=0.556 | 0.57 (0.15-2.17) P=0.409 |
| Headache disorder, N (%) | 3.66 (0.41-32.30) P=0.243 | 1.91 (0.20-17.85) P=0.571 | -- |
| Dementia, N (%) | 1.42 (0.34-5.98) P=0.629 | -- | 1.11 (0.26-4.71) P=0.883 |
| Psychiatric history, N (%) | 0.37 (0.10-1.33) P=0.127 | 4.21 (0.05-18.71) P=0.059 | 1.28 (0.35-4.66) P=0.711 |
| Neuro complication during index COVID-19 hospitalization, N (%) | 1.02 (0.49-2.11) P=0.961 | 0.89 (0.27-2.98) P=0.849 | 1.03 (0.48-2.22) P=0.943 |
| Mechanically ventilated during hospitalization for COVID-19, N (%) | **0.26 (0.12-0.55) P<0.001** | 1.68 (0.50-5.62) P=0.400 | **3.70 (1.64-8.37) P=0.002** |
| COVID-19 Vaccination, N (%) | 0.75 (0.34-1.64) P=0.466 | 1.44 (0.37-5.65) P=0.602 | 1.21 (0.52-2.79) P=0.663 |
| **Symptom Clusters** |  |  |  |
| Symptom Cluster 1, N (%) | **2.55 (1.10-5.89) P=0.029** | 0.18 (0.02-1.44) P=0.106 | 0.59 (0.25-1.41) P=0.234 |
| Symptom Cluster 2, N (%) | 0.66 (0.23-1.88) P=0.434 | **15.70 (4.14-59.70) P<0.001** | **0.12 (0.02-0.98) P=0.048** |
| Symptom Cluster 3, N (%) | 0.56 (0.27-1.18) P=0.127 | 0.36 (0.10-1.27) P=0.111 | **3.08 (1.33-7.13) P=0.009** |
| Number of PASC symptoms, N (%) | 0.94 (0.87-1.01) P=0.109 | **1.24 (1.12-1.38) P<0.001** | 0.93 (0.85-1.02) P=0.145 |
| Number of therapies received, median (IQR) | **0.49 (0.37-0.64) P<0.001** | **3.81 (2.03-7.15) P<0.001** | **1.21 (1.02-1.44) P=0.034** |
| Duration of symptoms, median (IQ | 0.90 (0.79-1.02) P=0.087 | 1.14 (0.80-1.45) P=0.284 | 1.08 (0.94-1.24) P=0.278 |
| **12-month Outcomes** |  |  |  |
| Improved with therapy*, N (%) | **0.06 (0.01-0.58) P=0.015** | --- | 7.4 (0.81-67.6) P=0.076 |
| 12-mo Barthel Index, median (IQR) | **1.05 (1.02-1.07) P=0.001** | 0.98 (0.96-1.01) P=0.147 | **0.97 (0.95-0.99) P=0.003** |
| 12-mo T-MoCA, median (IQR) | 0.94 (0.83-1.06) P=0.293 | 0.95 (0.79-1.15) P=0.624 | 1.11 (0.96-1.28) P=0.149 |
| 12-mo mRS, median (IQR) | **0.62 (0.47-0.82) P=0.001** | 1.22 (0.79-1.89) P=0.364 | **1.56 (1.17-2.10) P=0.003** |
| 12-mo NeuroQoL Anxiety, median (IQR) | 1.01 (0.97-1.05) P=0.690 | 1.05 (0.98-1.12) P=0.204 | 0.97 (0.93-1.02) P=0.203 |
| 12-mo NeuroQoL Depression, median (IQR) | 0.99 (0.95-1.04) P=0.704 | 1.05 (0.97-1.13) P=0.228 | 0.99 (0.94-1.04) P=0.687 |
| 12-mo NeuroQoL Fatigue, median (IQR) | 0.99 (0.95-1.02) P=0.442 | 1.04 (0.99-1.10) P=0.155 | 1.00 (0.96-1.04) P=0.902 |
| 12-mo NeuroQoL Sleep, median (IQR) | 1.01 (0.97-1.04) P=0.774 | 1.05 (0.99-1.11) P=0.110 | 0.97 (0.94-1.01) P=0.168 |

*among those who had symptoms and received at least one intervention, logistic regression analyses adjusted for severity of index COVID-19 (as assessed by requirement for invasive mechanical ventilation)

Relationship assessed using binary logistic regression analyses (odds ratio [OR], 95% confidence interval [CI], P values). Bold=significance with P<0.05. mRS=modified Rankin Scale; T-MoCA=telephone Montreal Cognitive Assessment, NeuroQoL=NIH Neurological Quality of Life patient reported outcomes.
