# Supplementary material for: Post-acute sequelae of COVID-19 symptom phenotypes and therapeutic strategies: A prospective, observational study
Source: PLoS One. 2022 Sep 29;17(9):e0275274. doi: 10.1371/journal.pone.0275274 (PMC9521913; doi:10.1371/journal.pone.0275274)
Supplement: S5 Table — (DOCX) [file pone.0275274.s005.docx]

**Supplemental Table 5.** Comparison of patients included in analyses to patients with missing data

|  | Included Subjects  (N=122) | Subjects with Missing Data (N=12) | P |
| --- | --- | --- | --- |
| **Demographics** |  |  |  |
| Age, median (IQR) | 64 (55-71) | 74 (66-87) | **0.002** |
| Sex (male), (%) | 62% | 50% | 0.540 |
| Race (white), (%) | 62% | 73% | 0.533 |
| Education level >12 years, (%) | 82% | 67% | 0.319 |
| **Stressors** |  |  |  |
| At least one stressor, (%) | 61% | 11% | **0.005** |
| Number of stressors, median (IQR) | 1 (0-2) | 0 (0) | **0.010** |
| Social Isolation, (%) | 17% | 0% | 0.353 |
| Financial Insecurity, (%) | 20% | 0% | 0.210 |
| Unemployment, (%) | 14% | 0% | 0.604 |
| Food Insecurity, (%) | 3% | 0% | 1.00 |
| Homelessness, (%) | 1% | 0% | 1.00 |
| Domestic violence, (%) | 0% | 0% | --- |
| Relationship problems in household, (%) | 7% | 0% | 1.00 |
| Education disruption, (%) | 3% | 0% | 1.00 |
| Increased caregiver responsibilities, (%) | 7% | 0% | 1.00 |
| Personal Illness, (%) | 30% | 11% | 0.446 |
| New Disability, (%) | 10% | 11% | 1.00 |
| Death of close contact, (%) | 13% | 0% | 0.600 |
| Illness of close contact, (%) | 12% | 0% | 0.596 |
| Lack of access to child care, (%) | 1% | 0% | 1.00 |
| Political conflict with close contacts, (%) | 7% | 0% | 1.00 |
| **Comorbidities** |  |  |  |
| Pre-COVID disability (mRS), median (IQR) | 0 (0-1) | 2 (0-3) | 0.163 |
| Hypertension, (%) | 37% | 42% | 0.762 |
| Diabetes, (%) | 26% | 17% | 0.730 |
| COPD/Asthma, (%) | 12% | 0% | 0.364 |
| Headache Disorder, (%) | 5% | 0% | 1.00 |
| Dementia, (%) | 7% | 8% | 1.00 |
| Psychiatric history, (%) | 9% | 8% | 1.00 |
| **Index COVID-19 Hospitalization** |  |  |  |
| Neuro complication, (%) | 44% | 25% | 0.236 |
| Mechanically ventilated, (%) | 47% | 25% | 0.225 |
| Worst Sequential Organ Failure Assessment (SOFA) score, median (IQR) | 4 (3-10) | 4 (2-6) | 0.405 |
| Lowest % oxygen saturation, median (IQR) | 85 (74-90) | 90 (78-92) | 0.200 |
| Lowest mean arterial blood pressure (mmHg), median (IQR) | 66 (55-76) | 70 (60-82) | 0.427 |
| Acute renal failure, (%) | 17% | 17% | 1.00 |
| **12-month Quantitative Metrics** |  |  |  |
| 12-mo Barthel Index, median (IQR) | 100 (90-100) | 95 (69-100) | 0.125 |
| 12-mo T-MoCA, median (IQR) | 18 (15-20) | --- | --- |
| 12-mo mRS, median (IQR) | 2 (1-4) | 4 (3-6) | **<0.001** |
| 12-mo NeuroQoL Anxiety, median T-score (IQR) | 51 (44-56) | 48 (42-51) | 0.320 |
| 12-mo NeuroQoL Depression, median T-score (IQR) | 47 (37-51) | 45 (37-48) | 0.628 |
| 12-mo NeuroQoL Fatigue, median T-score (IQR) | 48 (42-55) | 46 (38-58) | 0.676 |
| 12-mo NeuroQoL Sleep, median T-score (IQR) | 50 (39-57) | 47 (39-54) | 0.695 |
