# Supplementary figures and images for: Post-acute sequelae of COVID-19 symptom phenotypes and therapeutic strategies: A prospective, observational study
Source: PLoS One. 2022 Sep 29;17(9):e0275274. doi: 10.1371/journal.pone.0275274 (PMC9521913; doi:10.1371/journal.pone.0275274)

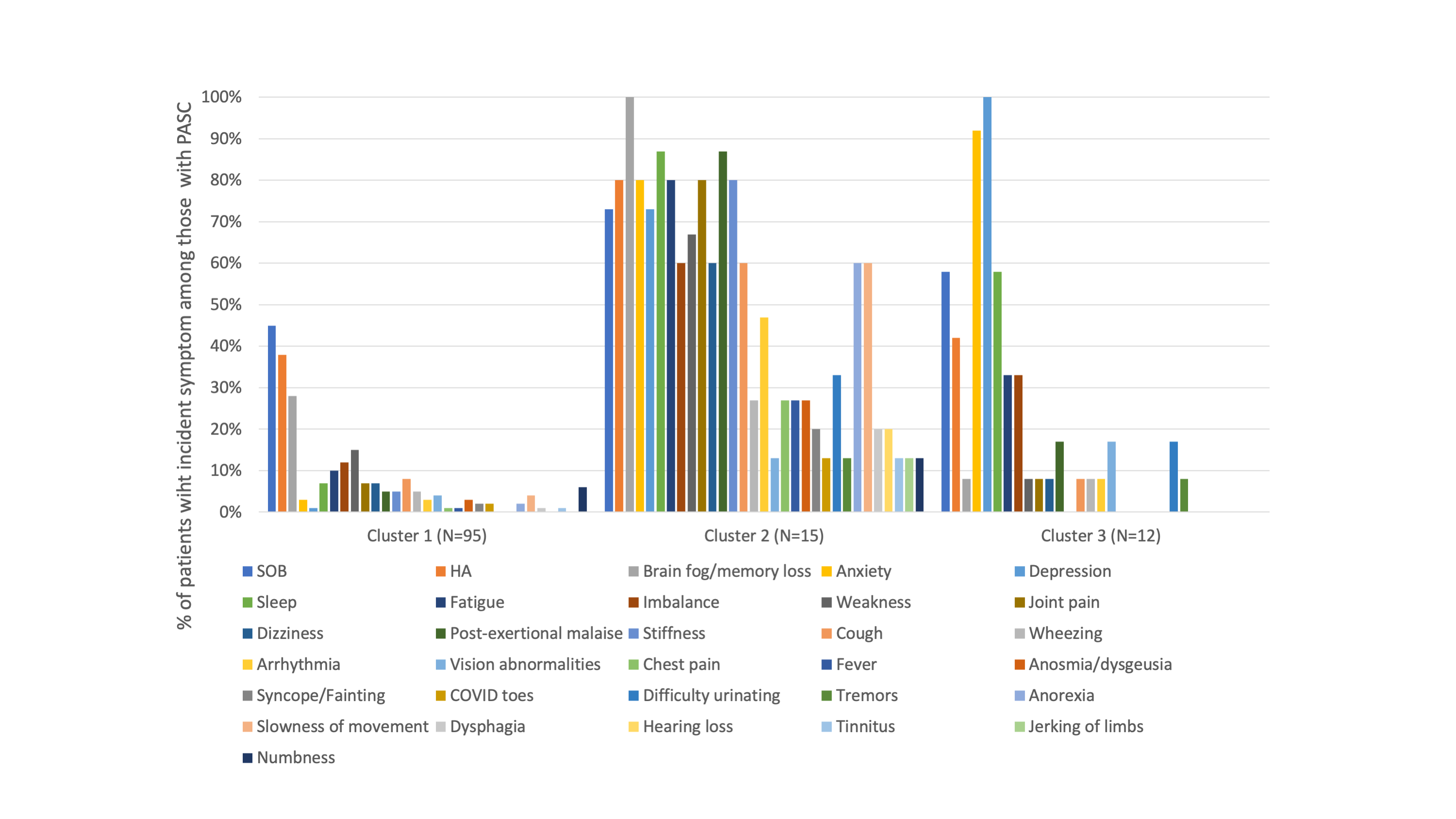

Supplement: S1 Fig — The three symptom clusters generated are similar to the primary analysis. (TIFF) [file pone.0275274.s006.tiff]
