# Supplementary material for: Post-acute sequelae of COVID-19 symptom phenotypes and therapeutic strategies: A prospective, observational study
Source: PLoS One. 2022 Sep 29;17(9):e0275274. doi: 10.1371/journal.pone.0275274 (PMC9521913; doi:10.1371/journal.pone.0275274)
Supplement: Attachment — Submitted filename: Reviewer 1 comments.docx [file pone.0275274.s008.docx]

I appreciate the improvements the authors have made on the manuscript. I have one concern on the following paragraph (sections highlighted in yellow).

1. Can the authors explain why the estimated aOR as reported in this paragraph lacks precision? Could this be a chance finding?
2. The last sentence that starts with “When ……. is incomplete!!!!

***Subjective Improvement among Therapy Groups***

Among patients who received at least one therapeutic intervention (N=85), those in Therapy Group B and C were significantly more likely to report improvement following therapy than Therapy Group A (100%, vs. 97% vs 83%, respectively, P=0.042). This was observed despite the fact that Therapy Group A had significantly better measures of activities of daily living (Barthel Index) and less severe disability (mRS) than other groups. After adjusting for severity of index COVID-19 illness (intubation status) and age, Therapy Group A was associated with significantly lower rates of subjective improvement compared to other therapy groups (adjusted OR 0.07, 95% CI 0.01-0.68, P=0.022). Conversely, belonging to Therapy Groups B or C (compared to A) was strongly associated with subjective improvement (aOR 14.5, 95% CI 1.5-142.9, P=0.022),

after adjusting for age and ventilator status. There was a significant association between the number of therapeutic interventions trialed and the subjective report of improvement following therapy (OR 3.00, 95% CI 1.22-7.35, P=0.017). When comparing

3. Can the authors justify the adequacy of the sample size to perform a cluster analysis of therapy groups? It seems the sample size used to perform the clustering was not adequate.
4. The authors should report briefly on those who received no therapy.
