# Supplementary material for: Post-acute sequelae of COVID-19 symptom phenotypes and therapeutic strategies: A prospective, observational study
Source: PLoS One. 2022 Sep 29;17(9):e0275274. doi: 10.1371/journal.pone.0275274 (PMC9521913; doi:10.1371/journal.pone.0275274)
Supplement: Attachment — Submitted filename: Response to Editor and Reviewers R2.docx [file pone.0275274.s009.docx]

Response to Editor and Reviewers R2

PONE-D-22-12045R1

Post-Acute Sequelae of COVID-19 Symptom Phenotypes and Therapeutic Strategies:  A Prospective, Observational Study

Reviewer 1:

I appreciate the improvements the authors have made on the manuscript. I have one concern on the following paragraph (sections highlighted in yellow).

1. Can the authors explain why the estimated aOR as reported in this paragraph lacks precision? Could this be a chance finding?

**We appreciate this query. When contrasting cluster A (n=72) to B-C combined (n=50), we detected large differences, with low probability that these were due to chance, despite relatively small sample sizes.  While the exact magnitude of difference may be imprecise, the likelihood of differences between these two groups is strong. Nonetheless, we agree that there is a wide confidence interval and the point estimate is imprecise. We have added the following to the limitations section of the discussion:**

**“Additionally, there were too few patients in certain treatment categories to make any statements regarding efficacy. While those in Therapy Group A reported lower rates of subjective improvement compared to Groups B or C, confidence intervals were wide suggesting imprecision in point estimates.” (page 12, paragraph 1)”**

2. The last sentence that starts with “When ……. is incomplete!!!!

**We thank the reviewer for catching this typo and have deleted it.**

***Subjective Improvement among Therapy Groups***

Among patients who received at least one therapeutic intervention (N=85), those in Therapy Group B and C were significantly more likely to report improvement following therapy than Therapy Group A (100%, vs. 97% vs 83%, respectively, P=0.042). This was observed despite the fact that Therapy Group A had significantly better measures of activities of daily living (Barthel Index) and less severe disability (mRS) than other groups. After adjusting for severity of index COVID-19 illness (intubation status) and age, Therapy Group A was associated with significantly lower rates of subjective improvement compared to other therapy groups (adjusted OR 0.07, 95% CI 0.01-0.68, P=0.022). Conversely, belonging to Therapy Groups B or C (compared to A) was strongly associated with subjective improvement (aOR 14.5, 95% CI 1.5-142.9, P=0.022),

after adjusting for age and ventilator status. There was a significant association between the number of therapeutic interventions trialed and the subjective report of improvement following therapy (OR 3.00, 95% CI 1.22-7.35, P=0.017). When comparing

3. Can the authors justify the adequacy of the sample size to perform a cluster analysis of therapy groups? It seems the sample size used to perform the clustering was not adequate.

**We appreciate this query. For cluster analyses, the goal is to determine whether subgroups exist within a sample vs a null hypothesis of a single group, and power is generally defined as the probability of correctly detecting that subgroups are present, which increases as a function of both ‘effect size’ (magnitude of difference between groups) and sample size. When separation between groups is large, small sample sizes are not a major concern.  Studies have shown that most algorithms can perform optimally even with lower separations with a minimum sample size of 20–30 observations per subgroup (Dalmaijer, E.S., Nord, C.L. & Astle, D.E. Statistical power for cluster analysis. *BMC Bioinformatics* 23, 205 (2022).** [**https://doi.org/10.1186/s12859-022-04675-1**](https://doi.org/10.1186/s12859-022-04675-1)**). In our case, we had sample sizes of 12-72 per cluster.  When contrasting cluster A (n=72) to B-C combined (n=50), we detected large differences, with low probability that these were due to chance, despite relatively small sample sizes.  While the exact magnitude of difference may be imprecise, the likelihood of differences between these two groups is strong.**

**We have added the above reference to the methods section (page 5, paragraph 2, reference 29)**

4. The authors should report briefly on those who received no therapy.

**The reviewer makes a good suggestion. We have added the following to the results section (page 8, paragraph 2):**

**“Among those who received no therapeutic interventions (N=37), the median age was 62 (IQR 51-72), 67% were male and 30% required mechanical ventilation during hospitalization. There were no significant differences in age or sex among those who did or did not receive a therapeutic intervention. However, those who did not receive any therapies were less likely to have required mechanical ventilation during index COVID-19 than those who received some intervention (30% versus 54%, P =0.018).”**
